# HOXA13 promotes gastric cancer progression partially via the FN1-mediated FAK/Src axis

**DOI:** 10.1186/s40164-022-00260-7

**Published:** 2022-02-23

**Authors:** Zhiwei Qin, Chongzhi Zhou

**Affiliations:** Department of General Surgery, Shanghai General Hospital, Shanghai Jiao Tong University School of Medicine, 85 Wujin Road, Shanghai, China

**Keywords:** Gastric cancer, HOXA13, FN1, FAK/Src, miR-449a

## Abstract

**Background:**

Gastric cancer (GC) is one of the most common cancers causing a poor prognosis worldwide. HOXA13, as a member of the homeobox (HOX) family, is involved in the regulation of cancer progression and has attracted increasing attention, as a potential novel target for anticancer strategies. However, the significance of HOXA13 in GC remains unclear. This article aims to explore the potential mechanism of HOXA13 in GC progression.

**Methods:**

Quantitative real-time PCR was carried out to detect the expression of HOXA13 and FN1 and the correlation between HOXA13 and FN1 in GC tissues. In vitro assays were conducted to investigate the role of HOXA13 and FN1 in the malignant phenotypes of GC cells and the function of HOXA13 in the activation of the FAK/Src axis in GC cells. Coimmunoprecipitation was performed to reveal the relationship between ITGA5, ITGB1 and FN1 in GC cells. A dual luciferase assay was performed to assess miR-449a-targeted regulation of HOXA13 expression.

**Results:**

Quantitative real-time PCR verified that HOXA13 was elevated and positively correlated with FN1 in GC. In vitro and in vivo assays demonstrated that high expression of HOXA13 promoted GC progression, especially metastasis. Mechanistically, rescue experiments, chromatin immunoprecipitation and dual luciferase assays revealed that HOXA13 directly bound to the FN1 promoter region to enhance the activation of the FAK/Src axis, leading to GC cell proliferation and metastasis. Furthermore, the result of a dual luciferase assay suggested that HOXA13 was directly targeted by miR-449a.

**Conclusions:**

Our results show that HOXA13 is a positive regulator of the FAK/Src axis mediated by FN1 in GC and promotes GC progression. Thus, targeting HOXA13, together with FN1, may provide a novel prospective anticancer strategy.

**Supplementary Information:**

The online version contains supplementary material available at 10.1186/s40164-022-00260-7.

## Background

HOX genes encode a family of highly evolutionarily conserved homeodomain-containing transcription factors [1, 2]. The dysregulation of HOX gene expression plays a key role in the regulation of tumorigenesis [3, 4]. As a member of the HOX gene family, HOXA13 has been specifically implicated as the critical factor of the progression of some cancers [5, 6]. Previously, we found that HOXA13 overexpression could promote the proliferation and tumorigenicity of GC cells, and signaling pathways, such as the PI3K-Akt, MAPK and mTOR signaling pathways, were involved in HOXA13-overexpressing GC cells [7]. However, the functional role HOXA13 plays and the underlying molecular mechanism in the progression of GC have not been elucidated in detail.

FAK/Src, a formed dual-kinase complex, is involved in the regulation of the PI3K-Akt signaling pathway and Erk signaling pathway, an important component of the MAPK signaling pathway [8, 9]. Fibronectin 1 (FN1) plays an important role in the activation of the FAK/Src axis. FN1 binds to transmembrane glycoprotein signaling receptors, especially integrin α5 (ITGA5) and integrin β1 (ITGB1) [10–13], and then increases the kinase activities of downstream targets, including the FAK/Src, Akt and Erk1/2 signaling cascades [8, 14]. FN1 could exert its function in different biological processes, and it could promote carcinogenesis in many cancers [15]. According to the above findings, we believe that it is worth further studying whether FN1 and the FAK/Src axis participate in the promotion of GC by HOXA13.

On this basis, we found that HOXA13 overexpression could promote proliferation and metastasis in GC cells, possibly by transcriptionally regulating FN1 and then activating the FAK/Src complex, causing phosphorylation of Akt and Erk1/2. Therefore, the present study provides a new potential effect of HOXA13 on modulating the progression of GC via the FAK/Src axis by regulating FN1.

## Methods

### Patients and tissue specimens

Human primary GC tissues and adjacent normal tissues used in this study were collected from GC patients who were treated in the Department of General Surgery of Shanghai General Hospital Affiliated to Shanghai Jiao Tong University (Shanghai, China). This study was approved by the Ethics Committee of Shanghai General Hospital.

### Bioinformatic analysis

The bioinformatic data were downloaded from Gene Expression Omnibus (GEO, http://www.ncbi.nlm.nih.gov/geo/). The JASPAR database (http://jaspar.genereg.net/) was used to predict potential binding positions of HOXA13 in the promoter region of FN1. The StarBase database (http://starbase.sysu.edu.cn/) and TargetScan database (http://www.targetscan.org/vert_72/) were used to retrieve the microRNAs (miRNAs) that regulate HOXA13.

### Quantitative real-time polymerase chain reaction (qRT-PCR)

Total RNA from GC cells and tissues was extracted using TRIzol (Takara, Shiga, Japan) according to the manufacturer’s instructions. HOXA13 and FN1 messenger RNA (mRNA) were detected as previously described [7]. For miR-449a detection, RNAs were reverse transcribed into complementary DNA (cDNA) using a HyperScript^TM^ III miRNA 1st Strand cDNA Synthesis Kit (EnzyArtisan Biotech, Shanghai, China). qRT-PCR was performed using 2× S6 Universal SYBR qPCR Mix (EnzyArtisan Biotech). The primer sequences used in qRT-PCR are listed in Additional file 3: Table S1. Each qRT-PCR was performed in triplicate, and the relative expression levels were normalized to GAPDH or U6 and calculated using the 2^−ΔΔCt^ method.

### Western blot analysis

Total proteins were extracted, and Western blot analysis was performed as previously described [7]. The primary antibodies used in Western blotting are listed in Additional file 3: Table S2.

### Immunohistochemical staining

Immunohistochemical staining was performed as previously described [7]. The detailed antibodies and their concentrations were as follows: anti-HOXA13 (1:200; Abcam) and anti-FN1 (1:100, Cell Signaling Technology).

### Coimmunoprecipitation (Co-IP)

Cell lysates were incubated with anti-FN1 (Cell Signaling Technology) or control IgG (Cell Signaling Technology) at 4 °C for 2 h, and then 20 µL Protein A/G PLUS-Agarose beads (Santa Cruz) were added and incubated at 4 °C overnight. Precipitates were washed three times with RIPA lysis buffer and detected by Western blotting.

### Cell culture, transfection, and generation of stable cell lines

The normal gastric epithelial cell line GES-1 and the human gastric cancer cell lines (AGS, MKN28, MKN45 and SGC-7901) were preserved by the Department of General Surgery of Shanghai General Hospital Affiliated to Shanghai Jiao Tong University. The cell lines were maintained in RPMI-1640 medium containing 10% FBS (Thermo Fisher Scientific, Waltham, MA, USA) and 1% penicillin–streptomycin (NCM Biotechnology, Jiangsu, China) at 37 °C under a humidified atmosphere containing 5% CO_2_.

FN1 overexpression plasmid, FN1 siRNA, miR-449a mimics and miR-449a inhibitor were all synthesized by Genomeditech (Shanghai, China). For transient transfection, proliferating cells in 6-well cell culture plates were incubated in serum-free medium containing plasmid, siRNA, mimics or inhibitor and Lipofectamine 2000 (Thermo Fisher Scientific) for 6 h. After that, the cells were incubated in complete medium for 48 h, followed by further experiments.

The HOXA13 lentiviral vector and HOXA13 shRNA lentiviral vector were obtained from Genomeditech. Lentiviruses were transfected into GC cells, and then stably transfected cells were selected with puromycin (InvivoGen, San Diego, CA, USA). Stable cell lines with luciferase were selected by blasticidin (InvivoGen). The cell lines were divided into the following categories: Mock, control group without any transfection; Ctrl, transfected with the lentiviral vector containing the control fragment or the control shRNA lentivirus; AGS-HOXA13-OV or MKN28-HOXA13-OV, transfected with the lentivirus containing the HOXA13 fragment; SGC-7901-sh-HOXA13 or MKN45-sh-HOXA13, transfected with HOXA13 lenti-shRNA.

### Cell growth assay

CCK-8 (Dojindo, Kumamoto, Japan) was used to generate cell growth curves and performed as previously described [7]. The DNA synthesis rate was detected by using a Cell-Light EdU DNA Cell Proliferation Kit (RiboBio, Guangdong, China). Cells (1 × 10^4^/well) were seeded into 96-well plates, cultured for 24 h, incubated with 50 µmol/L EdU solution for 2 h and fixed with 4% paraformaldehyde. After that, the cells were sealed with Apollo dye solution and Hoechst 33342. The percentage of EdU-stained cells to Hoechst-stained cells was generated to evaluate cell proliferation. These experiments were performed independently in triplicate.

### Flow cytometry analysis of cell apoptosis

The cell apoptosis assay was performed with the Annexin V-PE/7‐AAD Apoptosis Detection Kit (MultiSciences, Zhejiang, China). Cells were collected and resuspended in 500 µL 1× binding buffer and then stained with 5 µL Annexin V-PE and 10 µL 7-AAD for 15 min in the dark. Apoptotic cells were analyzed by flow cytometry (BD Biosciences, San Jose, CA, USA). The experiment was performed independently in triplicate.

### Conditioned mediums (CMs) collection

Cells were seeded in 6-well cell culture plates at 20 × 10^4^ cells per well. After 24 h, the cells were washed with serum-free medium three times and then mixed with 2 mL serum-free medium. After 24 h, the CMs were centrifuged to remove the cell debris, followed by further experiments.

### Tube formation assay

The 96-well cell culture plates were coated with Matrigel (Corning, New York, NY, USA) for 1 h at 37 °C, and then human umbilical vein endothelial cells (HUVECs) (2 × 10^4^/well) were suspended in CMs and seeded into 96-well cell culture plates. After 6 h, photographs were taken to count the number of tubes formed. The experiment was performed independently in triplicate.

### Cell migration and invasion assay

Cells were diluted to 20 × 10^4^/mL with serum-free medium, and 200 µL cell suspension was added to the upper chamber in a Transwell 24-well Boyden chamber (8.0 μm pore size; Corning). The experiment was performed as previously described [7]. The experiment was performed independently in triplicate.

### Nude mice metastatic models

Four-week-old male BALB/C nude mice were used to establish GC metastatic models in vivo (n = 3 for each group). For the lung metastatic models, 1 × 10^6^ cells were injected through the mouse tail vein. For the abdominal cavity metastatic models, 5 × 10^6^ cells were injected into the abdominal cavities of nude mice. The basic situations of nude mice, including the eating situation, mental state and growth, were observed throughout the experiment. Luciferase imaging was conducted to detect metastatic progression with an IVIS-100 system (Caliper Life Sciences, Hopkinton, MA, USA) after intraperitoneal injection of d-luciferin potassium salt (CSNpharm, Chicago, IL, USA). After 4 weeks, all mice were sacrificed, and the metastatic nodules were measured. All animal experiments were approved by the Animal Care Committee of Shanghai General Hospital.

### Construction of FN1 promoter reporter plasmids and mutagenesis

A 1905-bp fragment DNA containing FN1 5′ sequences from − 1807 to + 98 relative to the transcription initiation site was subcloned into the pGL3-basic vector (Promega, Madison, WI, USA) to construct the full-length reporter plasmid (WT), which contained multiple predicted HOXA13-binding sites (HBS). Mutation reporters for this plasmid (MT, #1, #2, #3, #4, #5 and #6) were then generated. All constructs were confirmed via DNA Sequencing.

### Dual luciferase assay

To detect the transcriptional regulation of FN1 by HOXA13, the human FN1 promoter reporter plasmid was transfected into HEK-293 cells together with the HOXA13-overexpression plasmid or control vector. To detect the targeted regulation of HOXA13 by miR-449a, HEK-293 cells were cotransfected with wild-type or mutant 3′-untranslated region (UTR) vectors and miR-449a mimics or control vector. The luciferase activity in these cells was normalized via cotransfection of a pTK-Renilla luciferase reporter (Genomeditech) containing a full-length Renilla luciferase gene, which was quantified using the dual luciferase assay system (Promega) 48 h after transfection. All experiments were performed independently in triplicate.

### Chromatin immunoprecipitation (ChIP) assay

The AGS cell line was transfected with the lentivirus vector PGMLV-4931 with 3× flag (Genomeditech). A ChIP assay was performed in exponentially growing cells using a ChIP assay kit (Cell Signaling Technology). Briefly, cells were cross-linked with 1% formaldehyde, lysed in SDS buffer and sonicated to fragment. Then, sheared chromatin was immunoprecipitated with anti-Flag (Cell Signaling Technology). Normal rabbit IgG (Cell Signaling Technology) was used as a negative control. The primers used in the ChIP assay are listed in Additional file 3: Table S3.

### Statistical analysis

Data analyses were performed using the SPSS 22.0 statistical software package (SPSS, Chicago, IL, USA). Relationships between variables were estimated using Pearson’s correlation. Other quantitative results are presented as the mean ± SD and were compared using the two-tailed Student’s *t*-test. For all tests, statistical significance was considered at *P* values < 0.05.

## Results

### HOXA13 expression was elevated in GC

**Fig. 1:**
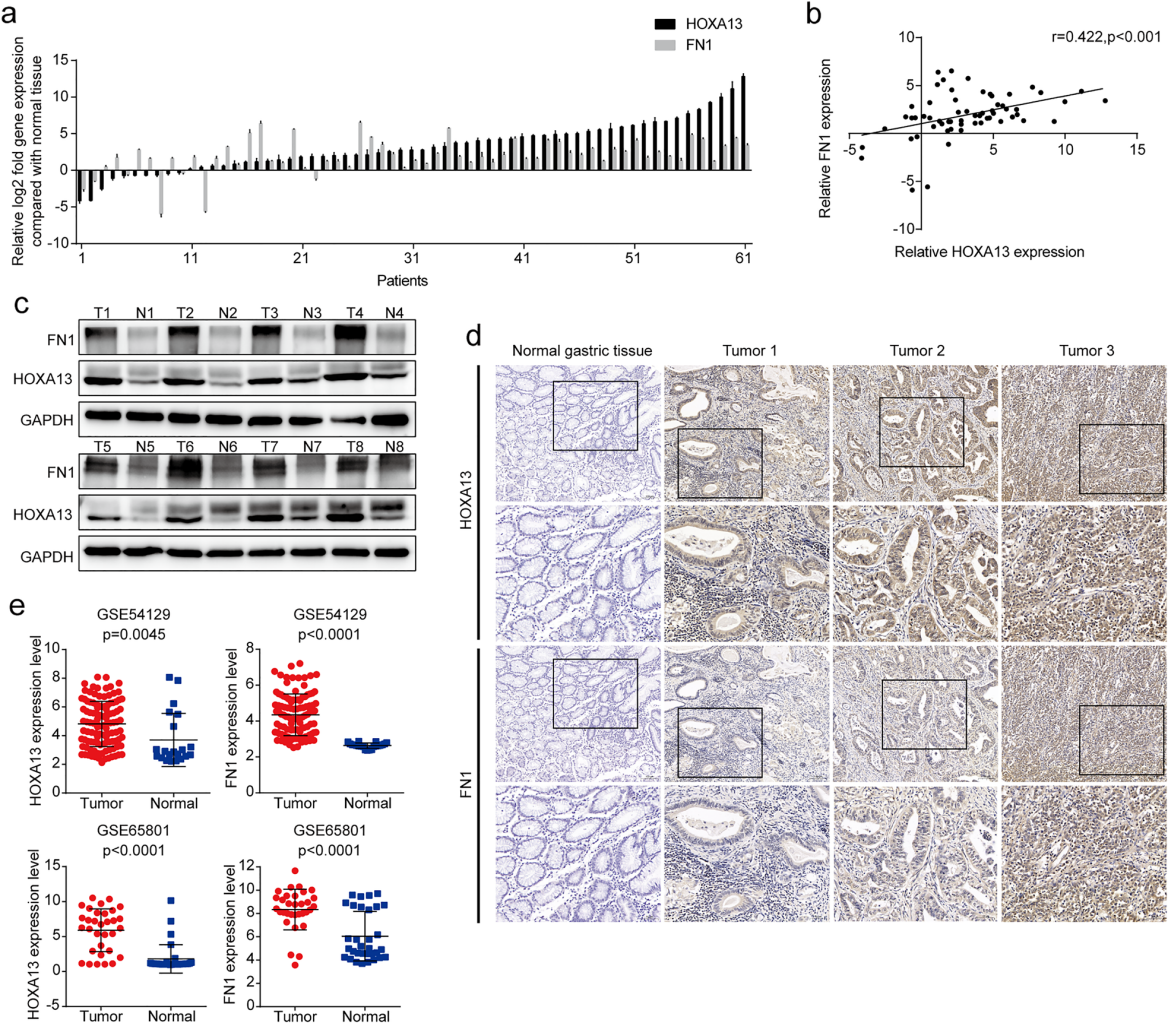
HOXA13 was upregulated and positively correlated with FN1 in GC tissues. **a** The mRNA expression of HOXA13 and FN1 in GC and adjacent normal tissues by qRT-PCR. **b** Pearson’s correlation between HOXA13 and FN1 based on the qRT-PCR results. **c** The protein expression of HOXA13 and FN1 in representative paired samples of GC and adjacent normal tissues by Western blot analysis. **d** Representative images of immunohistochemical staining with HOXA13 and FN1. Original magnification, ×100 (×200 for insert images). **e** The expression of HOXA13 and FN1 in GSE54129 and GSE65801 GC cohorts

The qRT-PCR results showed that HOXA13 was elevated in 83.61% (51/61) of GC tissues compared with adjacent normal tissues (Fig. 1a). Western blot analysis and immunohistochemical staining further confirmed that the protein expression level of HOXA13 was elevated in GC tissues (Fig. 1c, d). Similar results in which HOXA13 mRNA expression was elevated in GC were observed in the GSE54129 and GSE65801 databases (Fig. 1e).

### HOXA13 promoted growth and DNA synthesis of GC cells

**Fig. 2:**
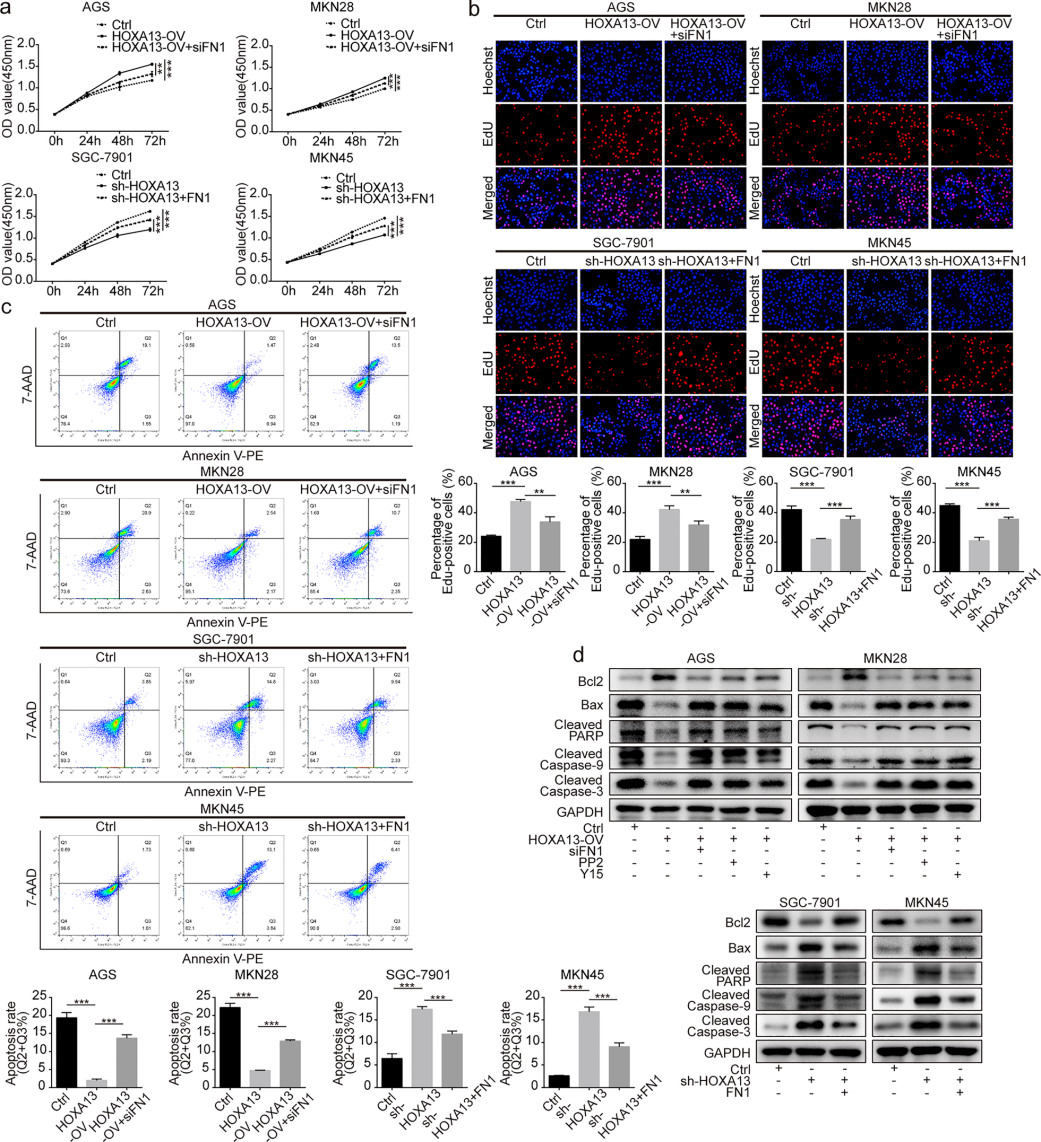
The effects of HOXA13 on proliferation and apoptosis in GC cells. **a** Cell proliferation ability was detected by CCK-8 assay in HOXA13 overexpression or knockdown stable cell lines and could be partly rescued by transfection with siFN1 or FN1 overexpression plasmid. **b** DNA synthesis of GC cells was tested by EdU staining assay in HOXA13 overexpression or knockdown stable cell lines and could be partly rescued by transfection with siFN1 or FN1 overexpression plasmid. **c** Cell apoptosis was examined by flow cytometry analysis in HOXA13 overexpression or knockdown stable cell lines and could be partly rescued by transfection with siFN1 or FN1 overexpression plasmid. **d** Bcl2, Bax, cleaved forms of Caspase-9, Caspase‐3, and PARP were detected by Western blot analysis (**p < 0.01, ***p < 0.001)

AGS and MKN28 cell lines were selected to generate stable HOXA13 overexpression cell lines, and SGC-7901 and MKN45 cell lines were selected to generate stable HOXA13 knockdown cell lines (Additional file 1: Fig. S1). The CCK-8 assay suggested that higher expression of HOXA13 could promote GC cell growth (Fig. 2a). The results of the EdU assay were consistent with the foregoing results (Fig. 2b).

### HOXA13 inhibited apoptosis in GC cells

Flow cytometry analysis of cell apoptosis showed that GC cells with higher expression of HOXA13 had a lower apoptosis ratio (Fig. 2c). Western blot analysis suggested that HOXA13 overexpression increased the expression of Bcl2 and decreased the expression of Bax, Cleaved Caspase-9, Cleaved Caspase-3, and Cleaved PARP, while HOXA13 knockdown caused the opposite alteration (Fig. 2d).

### HOXA13 promoted angiogenesis, migration and invasion ability of GC cells

**Fig. 3:**
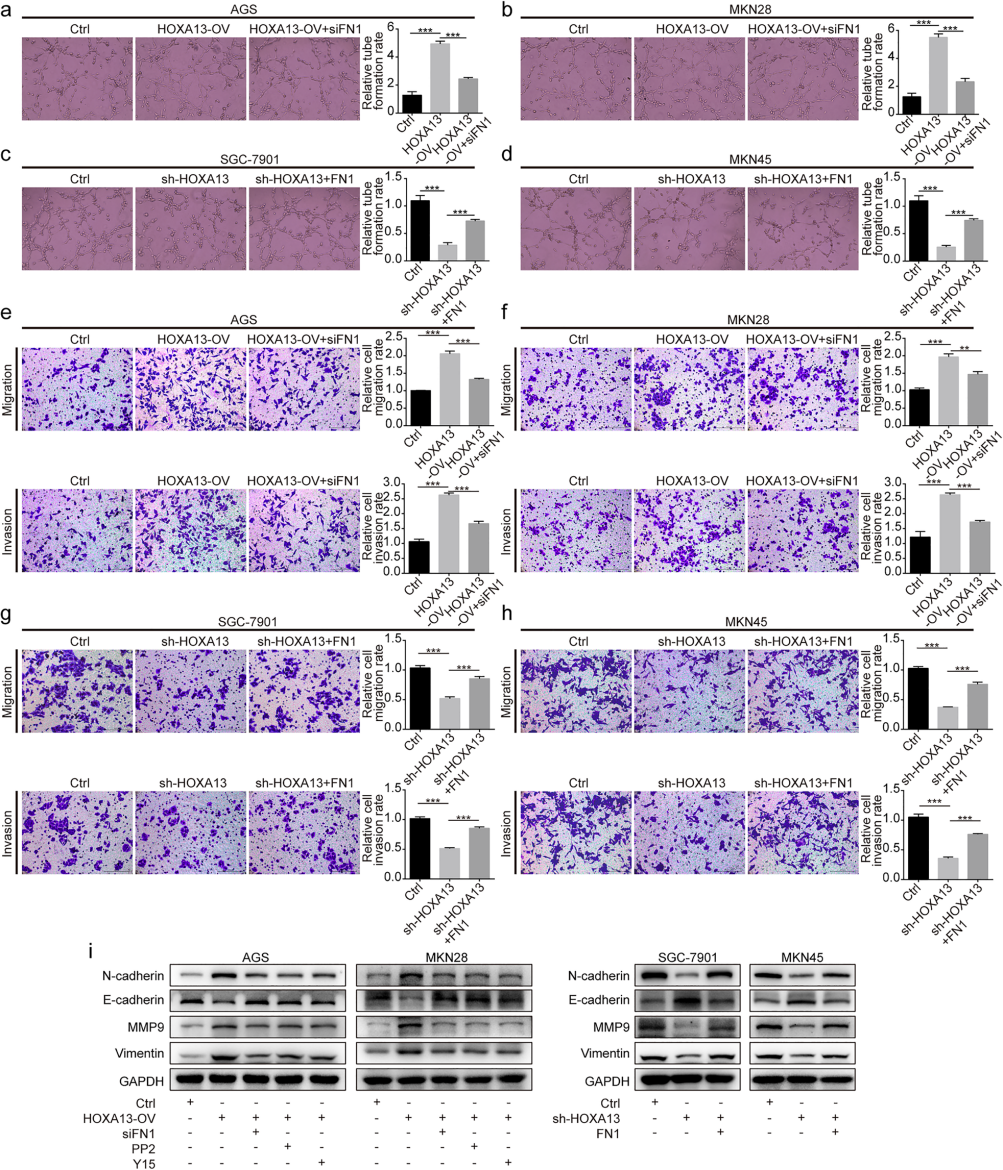
The effects of HOXA13 on angiogenesis, migration, and invasion in GC cells. **a**–**d** Angiogenesis ability of HOXA13 overexpression or knockdown stable cell lines was detected by tube formation assay and could be partly rescued by transfection with siFN1 or FN1 overexpression plasmid. Original magnification, ×200. **e**–**h** Migration and invasion ability of HOXA13 overexpression or knockdown stable cell lines were tested by Transwell assay and could be partly rescued by transfection with siFN1 or FN1 overexpression plasmid. Original magnification, ×200. **i** The protein expression of EMT-related markers was detected by Western blotting (**p < 0.01, ***p < 0.001)

Tube formation assays indicated that higher expression of HOXA13 increased the formation of tube-like structures (Fig. 3a–d). Transwell assays showed that HOXA13 overexpression or knockdown promoted or suppressed the migration and invasion of GC cells, respectively (Fig. 3e–h). Western blot analysis suggested that HOXA13 overexpression increased the expression of N-cadherin, Vimentin and MMP9 and decreased the expression of E-cadherin, while HOXA13 knockdown led to the opposite alteration (Fig. 3i).

### HOXA13 enhanced GC cell metastatic capacity in vivo

**Fig. 4:**
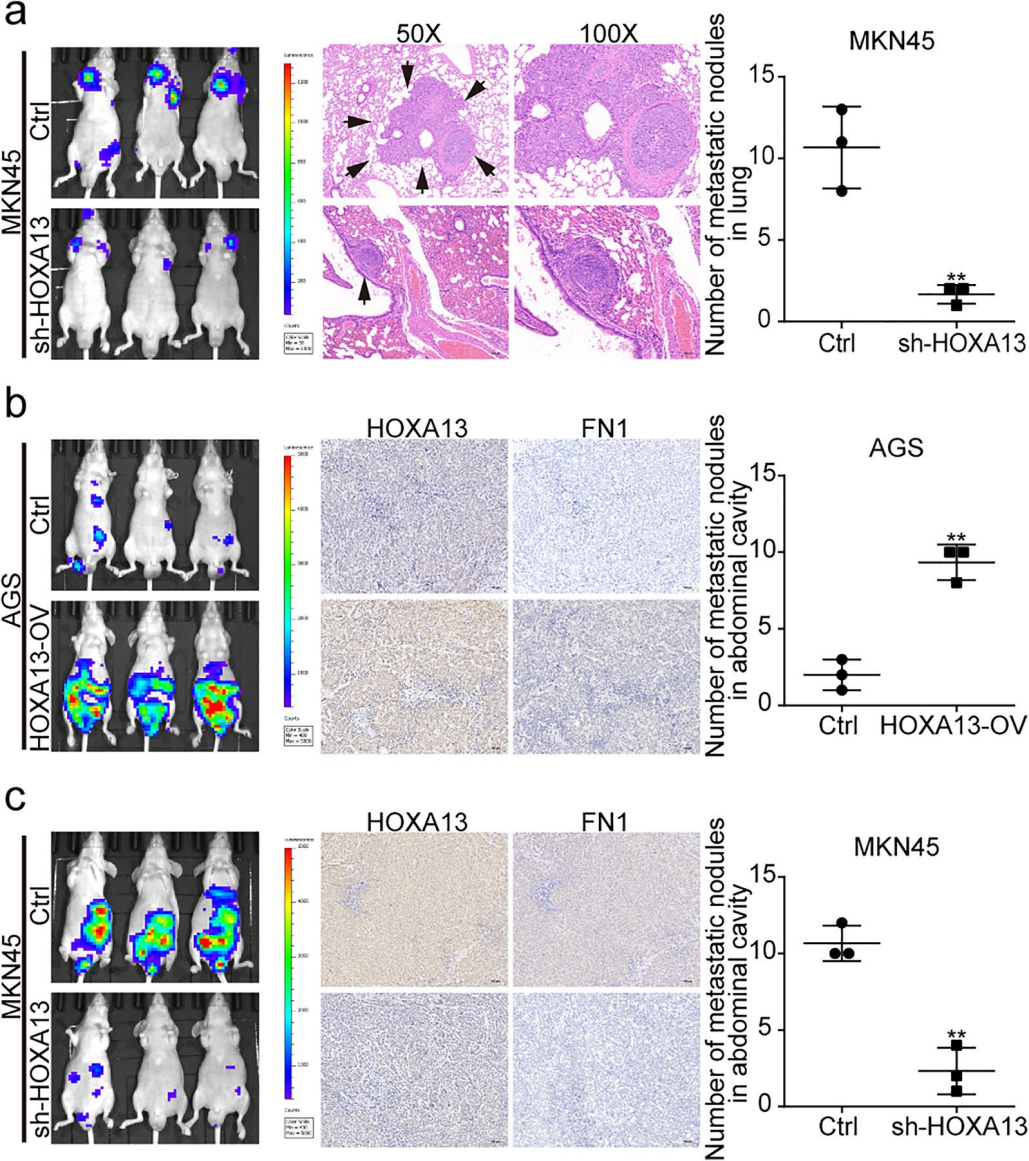
The effect of HOXA13 on metastasis in vivo. **a** Representative luciferase images of mice injected with MKN45-Ctrl or MKN45-sh-HOXA13 cells via the lateral tail veins and H&E staining in lungs. HOXA13 knockdown decreased the number of metastatic nodules in the lungs. **b** Representative luciferase images of mice injected with AGS-Ctrl or AGS-HOXA13-OV cells via the abdominal cavities and immunohistochemical staining in the nodules of abdominal cavities. HOXA13 overexpression increased the number of metastatic nodules in abdominal cavities. Original magnification, ×100. **c** Representative luciferase images of mice injected with MKN45-Ctrl or MKN45-sh-HOXA13 cells via the abdominal cavities and immunohistochemical staining in the nodules of abdominal cavities. HOXA13 knockdown decreased the number of metastatic nodules in abdominal cavities. Original magnification, ×100 (**p < 0.01)

To investigate the influence of HOXA13 on metastasis in vivo, we established lung and abdominal cavity metastatic models. The lung metastasis models showed that HOXA13 knockdown significantly reduced the number of metastatic lung nodules (Fig. 4a). For the abdominal cavity metastatic models, we observed that HOXA13 overexpression remarkably increased the number of metastatic nodules, while HOXA13 knockdown decreased the number of metastatic nodules (Fig. 4b, c). These results were consistent with the findings of the assays in vitro.

#### Elevated HOXA13 increased the phosphorylation levels of the FAK/Src axis

**Fig. 5:**
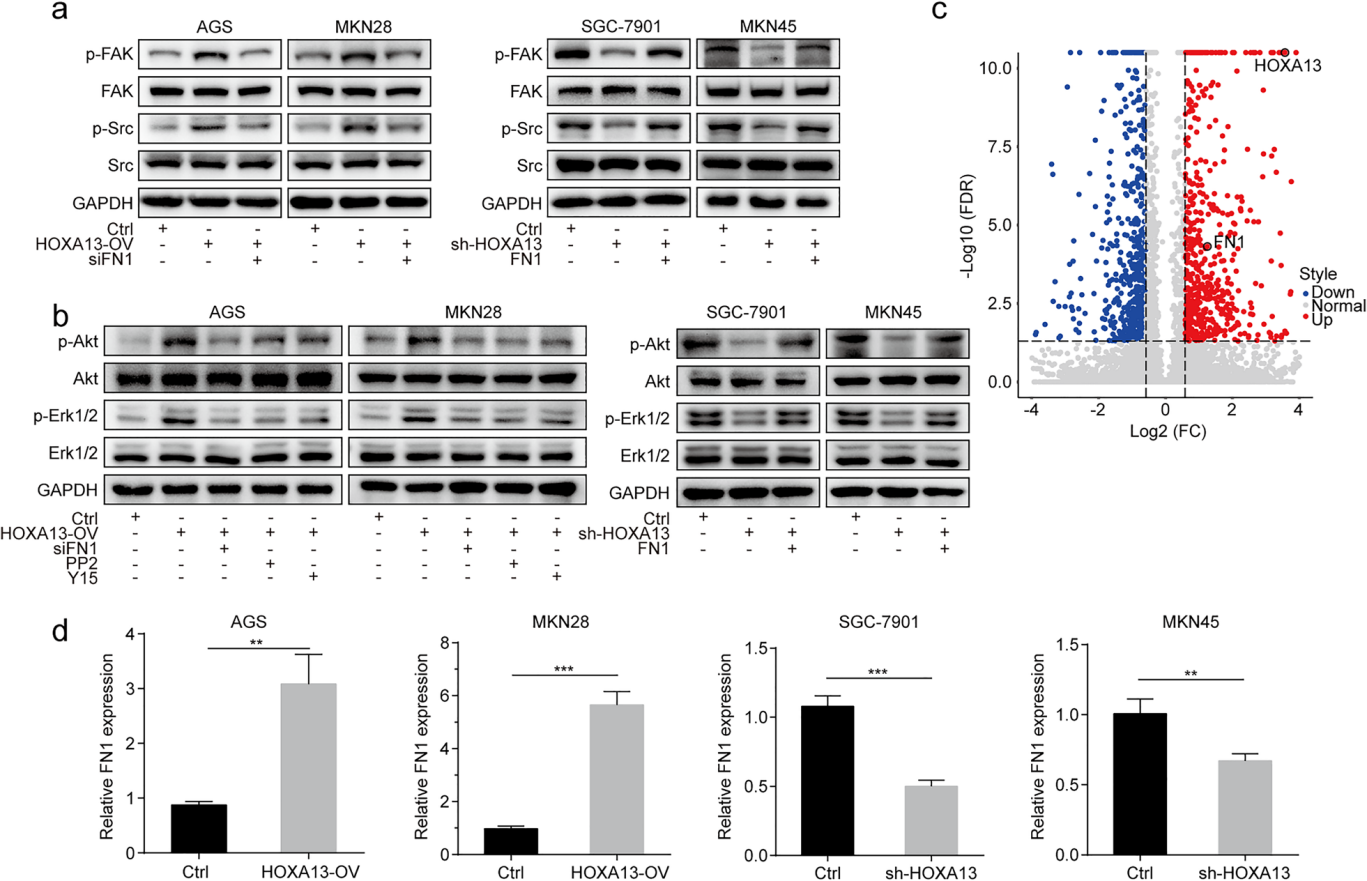
HOXA13 upregulated FN1 expression and activated the FAK/Src axis in GC. **a** HOXA13 overexpression stable cell lines were treated with siFN1, and HOXA13 knockdown stable cell lines were treated with FN1 overexpression plasmid, followed by Western blot analysis of the indicated proteins. **b** HOXA13 overexpression stable cell lines were treated with siFN1, PP2, or Y15, and HOXA13 knockdown stable cell lines were treated with FN1 overexpression plasmid, followed by Western blot analysis of the indicated proteins. **c** Volcano plot showing that FN1 was upregulated in AGS-HOXA13-OV cells. **d** The mRNA expression of FN1 in HOXA13 overexpression or knockdown stable cell lines was detected by qRT-PCR (**p < 0.01, ***p < 0.001)

As shown in Fig. 5a, b, Western blot analysis suggested that HOXA13 overexpression increased the phosphorylation levels of FAK, Src, Akt, and Erk1/2, while the shRNA-mediated knockdown of HOXA13 resulted in a decrease in the phosphorylation of the above markers. Considering the regulation of the FAK/Src axis in tumor progression, we speculated that the FAK/Src axis played an important role in the biological effects of HOXA13.

#### Elevated HOXA13 upregulated FN1 expression in GC

FN1 is a key molecule for the activation of the FAK/Src axis. We further analyzed the previous RNA-Seq transcriptome by volcano plot, which suggested that FN1 was upregulated in AGS-HOXA13-OV cells (Fig. 5c). Here, we observed that HOXA13 overexpression upregulated FN1 expression, while HOXA13 knockdown downregulated FN1 expression (Fig. 5d, Additional file 1: Fig. S1).

qRT-PCR showed that the expression of FN1 was elevated in 88.52% (54/61) of GC tissues compared with adjacent normal tissues (Fig. 1a). In addition, a statistically significant correlation between HOXA13 and FN1 expression was verified by Pearson’s correlation (r = 0.422, *p *< 0.001, Fig. 1b). Western blotting and immunohistochemical staining revealed elevated FN1 protein expression in GC tissues (Fig. 1c, d). Furthermore, GSE54129 and GSE65801 databases showed similar results: FN1 mRNA expression was elevated in GC (Fig. 1e).

Simultaneously, immunohistochemical staining was performed in the metastatic nodules of nude mice. The results showed that the expression of HOXA13 and FN1 was elevated in the AGS-HOXA13-OV group, but reduced in the MKN45-sh-HOXA13 group compared with the control group (Fig. 4b, c).

#### FN1 promoted malignant phenotypes in GC cells

**Fig. 6:**
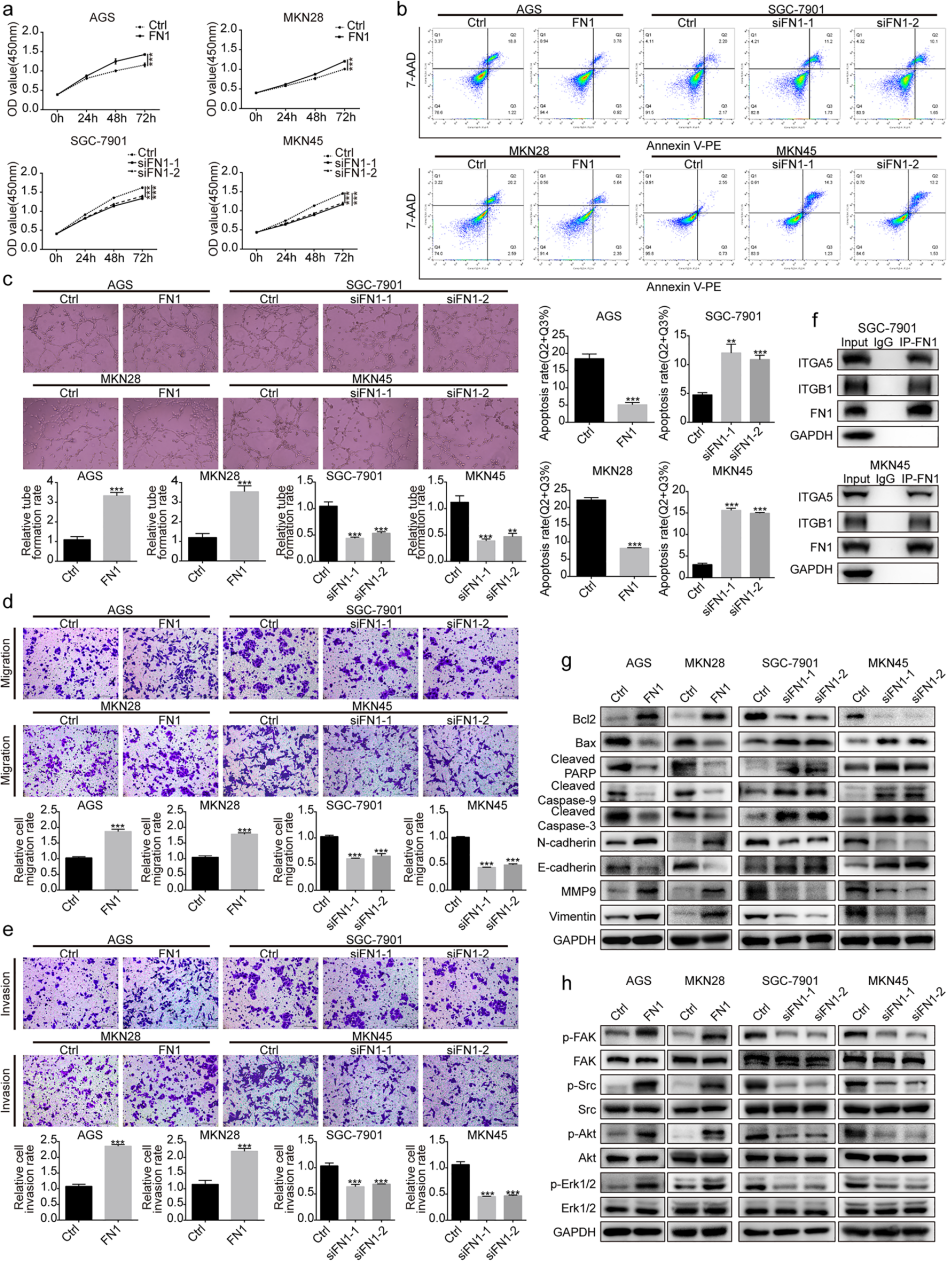
FN1 promoted malignant phenotypes in GC cells. **a** CCK-8 assay was used for GC cell proliferation. **b** Flow cytometry analysis was used to evaluate GC cell apoptosis. **c** Angiogenesis ability of GC cells was detected by tube formation assay. Original magnification, ×200. **d**, **e** Transwell assays were conducted to assess GC cell migration and invasion ability. Original magnification, ×200. **f** Whole SGC-7901 and MKN45 cell lysates were immunoprecipitated with antibodies against FN1 or IgG, followed by immunoblotting with antibodies against FN1, ITGA5, ITGB1 and GAPDH. **g** The protein expression of Bcl2, Bax, cleaved forms of Caspase-9, Caspase‐3, PARP and EMT-related markers was detected by Western blot analysis. **h** Western blot analysis of the FAK/Src axis-related proteins (**p < 0.01, ***p < 0.001)

To explore the effect of FN1 on the biological characteristics of GC cells, AGS and MKN28 cells transfected with FN1 overexpression plasmid, and SGC-7901 and MKN45 cells transfected with siRNA targeting FN1 were established (Additional file 2: Fig. S2). The CCK-8 assay showed a promotion or inhibition of cell proliferation in GC cells with FN1 upregulation or downregulation (Fig. 6a). Flow cytometry analysis showed that upregulation or downregulation of FN1 diminished or augmented the ratio of apoptotic cells, respectively (Fig. 6b). The formation of tube-like structures, migration and invasion was enhanced by upregulating FN1, while downregulation of FN1 led to the opposite result (Fig. 6c–e).

Western blot analysis revealed that upregulation of FN1 increased the expression of Bcl2, N-cadherin, Vimentin and MMP9 and decreased the expression of Bax, Cleaved Caspase-9, Cleaved Caspase-3, Cleaved PARP and E-cadherin, while downregulation of FN1 resulted in the opposite alteration (Fig. 6g).

#### Elevated FN1 activated the FAK/Src axis by phosphorylation

A previous study found that the binding of FN1 to ITGA5 and ITGB1 is a key step for activation of the FAK/Src axis [10]. The Co-IP experiment indicated that ITGA5 and ITGB1 were immunoprecipitated by FN1 antibody but not by the control IgG (Fig. 6f). Western blot analysis showed that FN1 upregulation increased FAK, Src, Erk1/2 and Akt phosphorylation, whereas FN1 downregulation showed the opposite alteration (Fig. 6h). Taken together, FN1 might exert its function in GC cells by interacting with ITGA5 and ITGB1 and then activating the FAK/Src axis.

#### Malignant phenotypes induced by HOXA13 were partly rescued by siFN1

To further investigate the relationship between HOXA13 and FN1, rescue experiments were performed. CCK-8, EdU and flow cytometry analysis suggested that the increased GC cell proliferation and lower ratio of apoptosis in HOXA13-overexpressing cells were partly impaired after transfection with siFN1, while the reduced GC cell proliferation and higher ratio of apoptosis in HOXA13-knockdown cells were partly reversed after transfection with the FN1 overexpression plasmid (Fig. 2a–c). Tube formation and Transwell assays indicated that siFN1 treatment partly rescued the increased formation of tube-like structures and the promotion of migration and invasion by HOXA13 overexpression, while the FN1 overexpression plasmid partly rescued the reduced formation of tube-like structures and the suppression of migration and invasion by HOXA13 knockdown (Fig. 3a–h).

Subsequently, Western blot analysis suggested that the increased expression of Bcl2, N-cadherin, Vimentin and MMP9 and the decreased expression of E-cadherin, Bax, Cleaved Caspase-9, Cleaved Caspase-3 and Cleaved PARP in HOXA13-overexpressing cells could be partly rescued after treatment with siFN1, PP2 (the Src inhibitor), or Y15 (the FAK inhibitor), while the opposite alteration was observed in HOXA13-knockdown cells transfected with the FN1 overexpression plasmid (Figs. 2d and 3i).

Next, we found that siFN1 could suppress the FAK/Src axis activation induced by HOXA13 overexpression, and FN1 overexpression plasmid could reverse the decreased phosphorylation levels of these markers caused by HOXA13 knockdown. Additionally, PP2 and Y15 suppressed Akt and Erk phosphorylation mediated by HOXA13 overexpression (Fig. 5a, b). These data indicated that HOXA13 induced cell proliferation, angiogenesis, migration and invasion in GC cells partly via the FAK/Src axis by regulating FN1.

#### HOXA13 upregulated FN1 expression by directly binding to its promoter

**Fig. 7:**
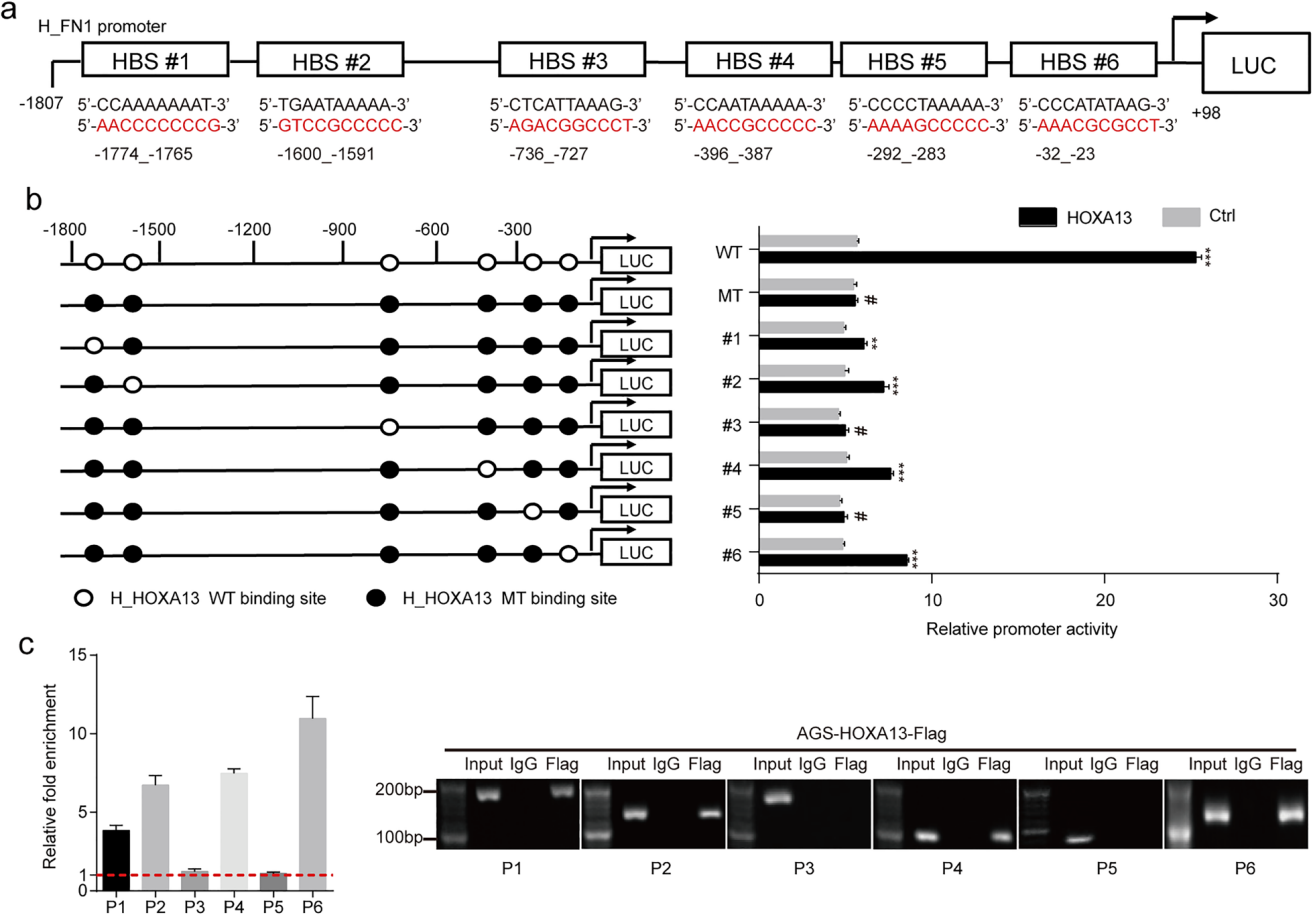
Direct binding of HOXA13 to the FN1 promoter region. **a** Six possible HBSs located at different sites in the FN1 promoter region. **b** Dual luciferase assay was conducted to examine FN1 promoter activity. **c** ChIP assays were performed with a specific anti-Flag antibody or IgG to determine the binding sites (**p < 0.01, ***p < 0.001, ^#^*p *> 0.05)

Six possible binding sites analyzed by the JASPAR database were selected for verification (Fig. 7a). According to the predicted binding sites, wild-type and mutation reporters (WT, MT, #1, #2, #3, #4, #5 and #6) were generated and tested by the dual luciferase assay. We found that the luciferase activity from WT, #1, #2, #4 and #6, but not the activity from MT, #3 and #5, was strongly stimulated when luciferase was cotransfected with HOXA13 (Fig. 7b). These results demonstrated that the regions of − 1774_− 1765, − 1600_− 1591, − 396_− 387 and − 32_− 23 might be important HOXA13 response elements in the FN1 promoter. On the basis of prediction, we designed and synthesized the corresponding primers for the ChIP assay. The results showed that HOXA13 had a higher enrichment in P1, P2, P4 and P6 than in P3 and P5 (Fig. 7c). In summary, these results suggested that HOXA13 directly bound to the region of the FN1 promoter to regulate its transcription.

#### HOXA13 was targeted by miR-449a in GC cells

**Fig. 8:**
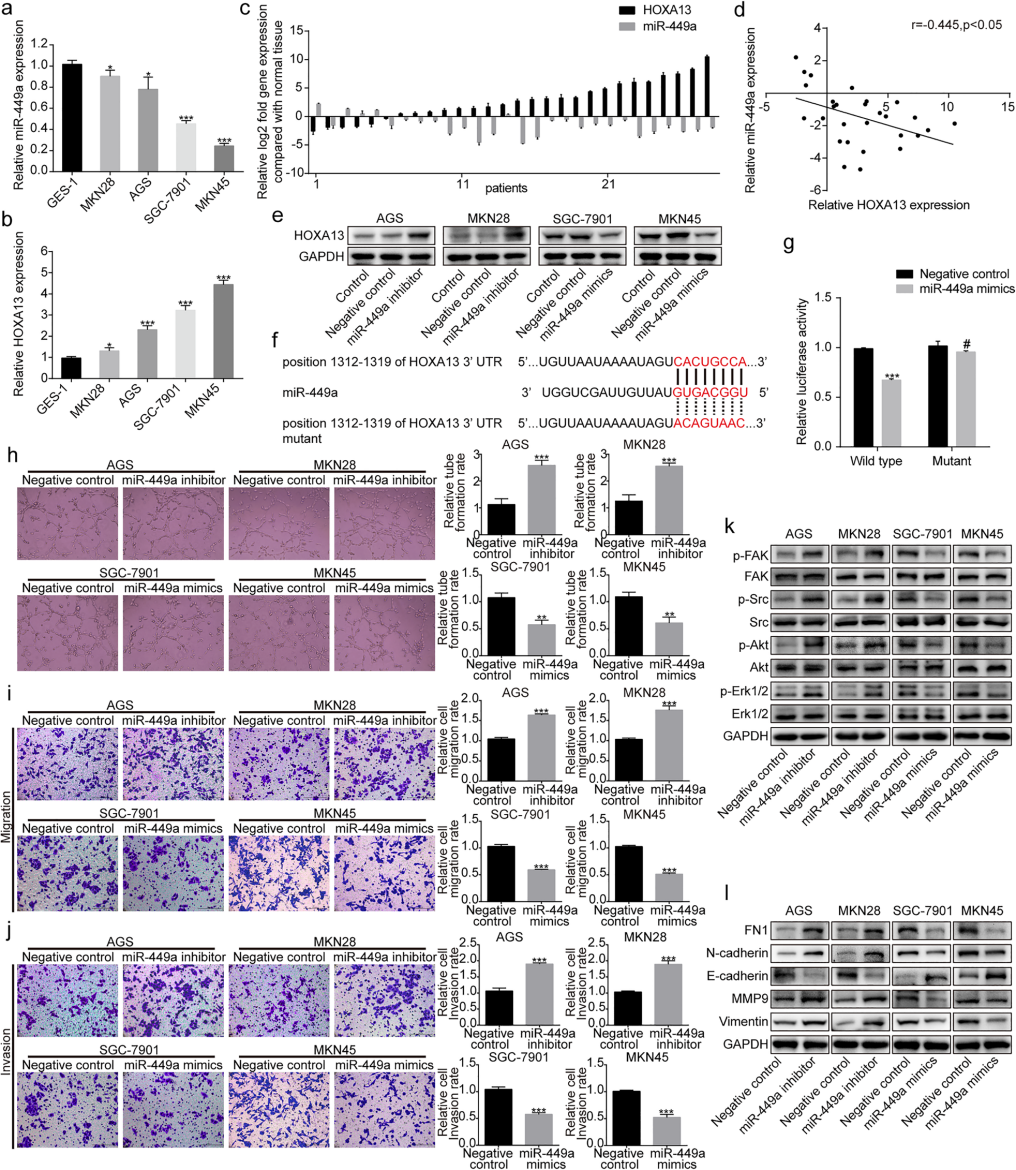
MiR-449a targeted regulation of HOXA13 expression. **a** Relative miR-449a expression in the normal gastric epithelial cell line GES-1 and GC cell lines by qRT-PCR. **b** Relative HOXA13 expression in the normal gastric epithelial cell line GES-1 and GC cell lines by qRT-PCR. **c** The expression of miR-449a and HOXA13 in GC and adjacent normal tissues by qRT-PCR. **d** Pearson’s correlation between miR-449a and HOXA13 based on the qRT-PCR results. **e** The regulation of HOXA13 expression produced by miR-449a. **f** Predicted binding sites between the HOXA13 3′-UTR and miR-449a. **g** Relative luciferase activity of the predicted wild-type or mutant binding site of miR-449a in the 3′-UTR of HOXA13 mRNA. **h** The angiogenic ability of GC cells was detected by tube formation assay. Original magnification, ×200. **i**, **j** Transwell assays were conducted to assess GC cell migration and invasion ability. Original magnification, ×200. **k** The expression of FAK/Src axis-related proteins in GC cells after transfection with miR-449a mimics or inhibitor was detected by Western blotting. **l** The protein expression of EMT-related markers in GC cells after transfection with miR-449a mimics or inhibitor was detected by Western blotting (*p < 0.05, **p < 0.01, ***p < 0.001, ^#^*p *> 0.05)

The StarBase database and TargetScan database were used to predict miRNAs that might regulate HOXA13 expression. Among the predicted miRNAs, miR-449a has been demonstrated to be related to GC progression [16, 17]. We therefore focused on whether miR-449a could regulate HOXA13. qRT-PCR showed that the expression of miR-449a was lower, while the expression of HOXA13 was higher in GC cells than in GES-1 normal gastric epithelial cells (Fig. 8a, b). We performed qRT-PCR in another 28 paired GC and adjacent normal tissues, which showed that miR-449a was reduced in 82.14% (23/28) of GC tissues (Fig. 8c). Additionally, a statistically negative correlation between miR-449a and HOXA13 expression was tested by Pearson’s correlation (r= − 0.445, *p *< 0.05, Fig. 8d). Western blot analysis revealed that HOXA13 expression was upregulated by transfection with the miR-449a inhibitor, while overexpression of miR-449a by transfection with miR-449a mimics markedly reduced the expression of HOXA13 (Fig. 8e). The predicted binding sites between the HOXA13 3′-UTR and miR-449a are shown in Fig. 8f. Dual luciferase assays showed that overexpression of miR-449a suppressed the luciferase activity of the wild-type luciferase constructs but not the mutant constructs in HEK-293 cells (Fig. 8g). Tube formation and Transwell assays indicated that the miR-449a inhibitor increased the formation of tube-like structures and promoted the migration and invasion of GC cells, while miR-449a mimics led to the opposite alteration (Fig. 8h–j). Next, we performed Western blotting to detect biomarkers related to the FAK/Src axis and the EMT process with altered miR-449a expression. As shown in Fig. 8k, the miR-449a inhibitor increased FAK, Src, Erk1/2 and Akt phosphorylation, whereas miR-449a mimics showed the opposite alteration. FN1, N-cadherin, Vimentin and MMP9 were upregulated, while E-cadherin was downregulated in GC cells transfected with miR-449a inhibitor. When cells were transfected with miR-449a mimics, the opposite results were observed (Fig. 8l). All these data demonstrated that miR-449a could inhibit GC malignant phenotypes and negatively regulate HOXA13 in GC cells.

## Discussion

It has been reported that the deregulated expression of HOX genes is associated with tumorigenesis [18, 19]. HOXA13, as a member of the HOX gene family, has been frequently researched in cancer progression [5, 6]. Here, we explored the potential mechanism of HOXA13 in GC progression.

In the current study, we found that the expression of HOXA13 was elevated in GC tissues compared with adjacent normal tissues. Then, through in vitro experiments, we found that HOXA13 could promote the malignant phenotypes of GC cells. By establishing GC metastatic models in vivo, we found that elevated HOXA13 increased metastatic nodules in nude mice. Our previous study showed that the MAPK and PI3K-Akt signaling pathways were engaged in GC progression induced by HOXA13 overexpression [7]. The MAPK and PI3K/Akt signaling pathways are involved in the regulation of many fundamental cellular processes, including cell proliferation, differentiation and survival, as well as in various aspects of oncogenesis, such as apoptosis, angiogenesis and metastasis [20, 21]. In the field of tumor research, increasing evidence has indicated that Erk signaling, as an important component of MAPK, is involved in cell proliferation, survival and metastasis [22]. Our previous study showed that Erk signaling might play an important role in the malignant phenotypes of GC cells induced by HOXA13 [7]. More noteworthy, current studies have shown that the FAK/Src complex plays a regulatory role in the Erk and PI3K-Akt signaling pathways [8, 9]. Therefore, further research should examine whether HOXA13 can activate the Erk and PI3K-Akt signaling pathways through the FAK/Src complex to promote GC progression. Western blot analysis showed that HOXA13 overexpression increased the phosphorylation levels of FAK, Src, Erk1/2, and Akt, which suggested that the FAK/Src axis played an important role in GC progression induced by HOXA13.

To elaborate the underlying mechanism in detail, we performed volcano plots to further analyze the previous RNA-Seq transcriptome. Significantly, a volcano plot showed that FN1 was elevated in AGS-HOXA13-OV cells, which was verified by Western blotting and qRT-PCR. Previous studies have shown that FN1 could promote cancer cell metastasis, angiogenesis and proliferation [23–25]. Most importantly, as a ligand glycoprotein, FN1 could bind to ITGA5 and ITGB1, resulting in recruitment and activation of signaling pathway-related proteins, such as the FAK/Src complex [10, 26]. Then, downstream targets, including Erk1/2 and Akt, could be activated [8, 9]. In our study, in vitro experiments showed that FN1 upregulation promoted malignant phenotypes in GC cells. By conducting Co-IP and Western blotting, we found that FN1 interacted with ITGA5 and ITGB1 and activated the FAK/Src axis.

Notably, a significant positive correlation between HOXA13 and FN1 expression was tested by Pearson’s correlation. To further explore whether HOXA13 promoted GC progression through FN1, we carried out rescue experiments, which showed that the positive or negative regulation of GC cells induced by HOXA13 overexpression or knockdown could be partly rescued by siFN1 or FN1 overexpression plasmids. Western blot analysis showed that siFN1 partly suppressed the FAK/Src axis activation induced by HOXA13 overexpression, and FN1 overexpression plasmid reversed the decreased phosphorylation levels of these markers caused by HOXA13 knockdown. Thus, we inferred that HOXA13 induced GC progression partly via the FAK/Src axis by regulating FN1.

As a transcription factor, could HOXA13 affect the expression of FN1 through transcriptional regulation? By searching the JASPAR database, we obtained several possible binding sites, suggesting that HOXA13 might be the transcription regulator of FN1. Dual luciferase assays demonstrated that the regions of − 1774_− 1765, − 1600_− 1591, − 396_− 387 and − 32_− 23 in the FN1 promoter might be the binding sites of HOXA13. ChIP assays showed that HOXA13 had a higher enrichment in the P1, P2, P4 and P6 areas than in the P3 and P5 areas. The results indicated that HOXA13 could directly bind to the region of the FN1 promoter to regulate its transcription.

MiRNAs are single-stranded, small noncoding RNA molecules that function by recognizing complementary sites in the 3′-UTR of target mRNAs [27, 28]. The dysregulation of miRNAs can interfere with the expression of downstream oncogenic or tumor-suppressive target genes, which are related to the pathogenesis of cancers [29]. It has been reported that miRNAs are involved in regulating EMT-related signaling pathways or EMT-inducing transcription factors to mediate the EMT process [30]. Therefore, we conducted preliminary research on the upstream regulatory mechanism of HOXA13 to explore whether miRNAs were involved in the regulation of HOXA13. The bioinformatic prediction results demonstrated that HOXA13 could be potentially regulated by miR-449a. The dysregulation of miR-449a exists in some types of cancers [31, 32]. We found that the expression of miR-449a was downregulated in GC cells and tissues and negatively correlated with HOXA13 expression. Dual luciferase assays and Western blotting revealed that miR-449a targeted the complementary site in the 3′-UTR of HOXA13 to decrease HOXA13 expression. Next, in vitro assays showed that miR-449a could suppress the angiogenesis, migration and invasion of GC cells and inhibit the FAK/Src axis activation.

**Fig. 9:**
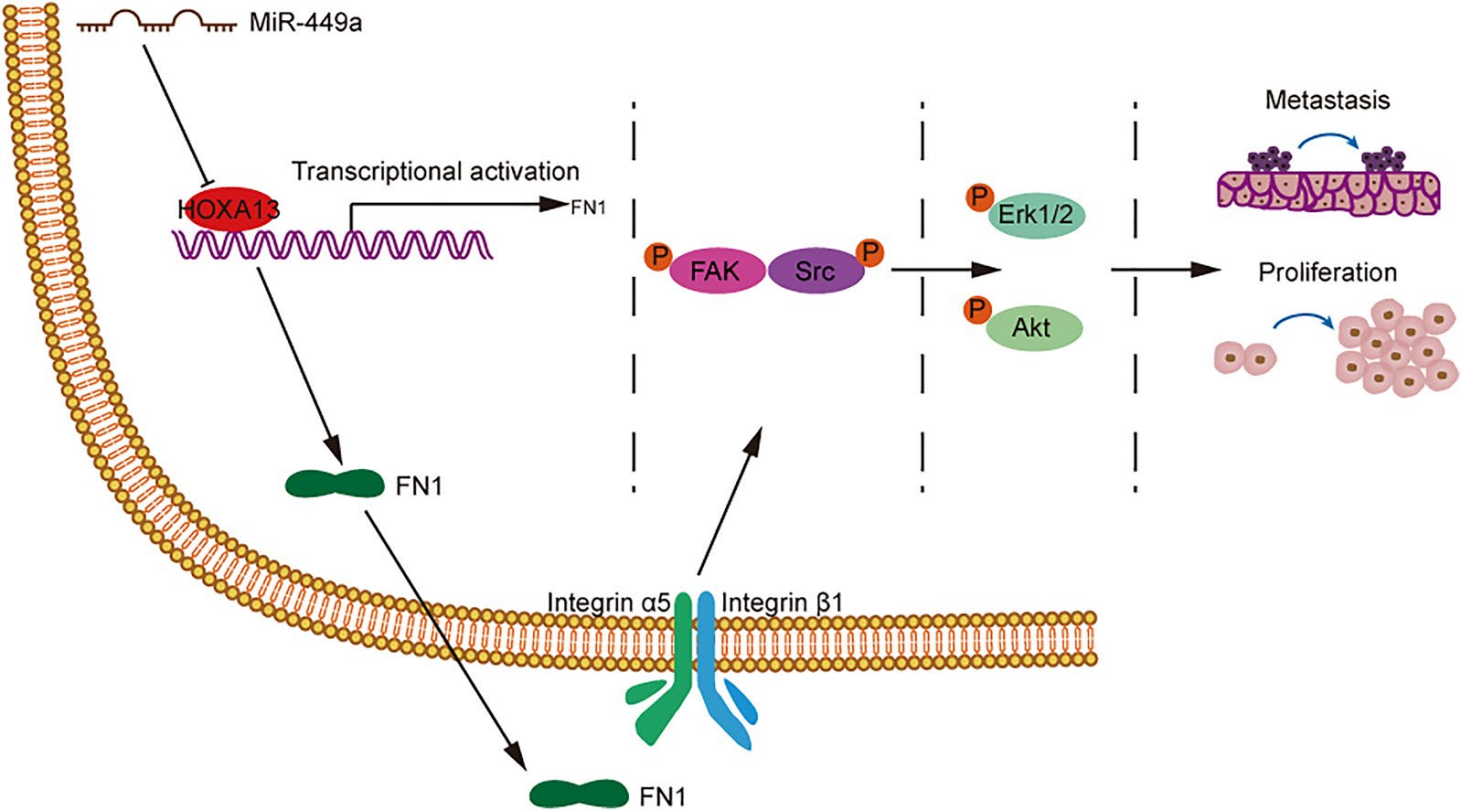
A possible schema as discussed was proposed. The elevation of HOXA13, which was related to the downregulation of miR-449a, regulated FN1 at the transcriptional level, thereby activating the FAK/Src axis to cause the proliferation and metastasis of GC

Overall, these data indicated that the upregulation of HOXA13, which might be related to downregulated miR-449a expression, transcriptionally regulated FN1 expression to promote the proliferation and metastasis process of GC through the FAK/Src axis (Fig. 9).

## Conclusions

Collectively, this study preliminarily revealed that elevated HOXA13, possibly regulated by loss of miR-449a, promoted the progression of GC via the FAK/Src axis by transcriptionally regulating FN1. Our findings demonstrated that HOXA13, together with FN1, might be a promising target for novel therapeutic strategies against GC.

## Supplementary Information

**Additional file 1: Figure S1. **The protein expression of HOXA13 and FN1 in HOXA13 overexpression or knockdown stable cell lines was verified by Western blotting.

**Additional file 2: Figure S2. **FN1 overexpression plasmid or siRNA targeting FN1 was transfected into GC cells. **a**, **b** AGS and MKN28 cells were transfected with FN1 overexpression plasmid. **c**, **d** SGC-7901 and MKN45 cells were transfected with siFN1-1 and siFN1-2 (**p < 0.01, ***p < 0.001).

**Additional file 3: Table S1.** Primer sequences for qRT-PCR. **Table S2.** Primary antibodies for Western blot analysis. **Table S3.** Primer sequences for ChIP assay.

## Data Availability

All data generated or analyzed during this study are included in this published article and its Additional files. Data from the publicly available datasets used in this study can be accessed at: GEO (http://www.ncbi.nlm.nih.gov/geo/), JASPAR (http://jaspar.genereg.net/), StarBase (http://starbase.sysu.edu.cn/), TargetScan (http://www.targetscan.org/vert_72/).

## Abbreviations

GC: Gastric cancer
HOXA13: Homeobox A13
FN1: Fibronectin 1
ITGA5: Integrin α5
ITGB1: Integrin β1
FAK: Focal adhesion kinase
Erk: Extracellular signal-regulated kinase
MAPK: Mitogen-activated protein kinase
PI3K: Phosphoinositide 3-kinase
EMT: Epithelial to mesenchymal transition
GEO: Gene Expression Omnibus
qRT-PCR: Quantitative real-time polymerase chain reaction
MMP9: Matrix metalloproteinase 9
PARP: Poly(ADP-ribose) polymerase
Co-IP: Coimmunoprecipitation
CCK-8: Cell Counting Kit-8
CMs: Conditioned mediums
HUVECs: Human umbilical vein endothelial cells
ChIP: Chromatin immunoprecipitation
