# Supplementary figures and images for: HOXA13 promotes gastric cancer progression partially via the FN1-mediated FAK/Src axis
Source: Exp Hematol Oncol. 2022 Feb 23;11:7. doi: 10.1186/s40164-022-00260-7 (PMC8864865; doi:10.1186/s40164-022-00260-7)

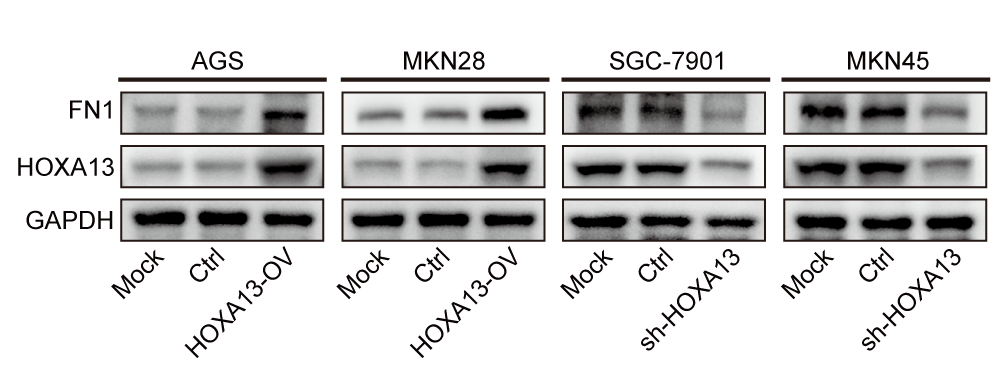

Supplement: Supplementary file 1 — Additional file 1: Figure S1. The protein expression of HOXA13 and FN1 in HOXA13 overexpression or knockdown stable cell lines was verified by Western blotting. [file 40164_2022_260_MOESM1_ESM.tif]

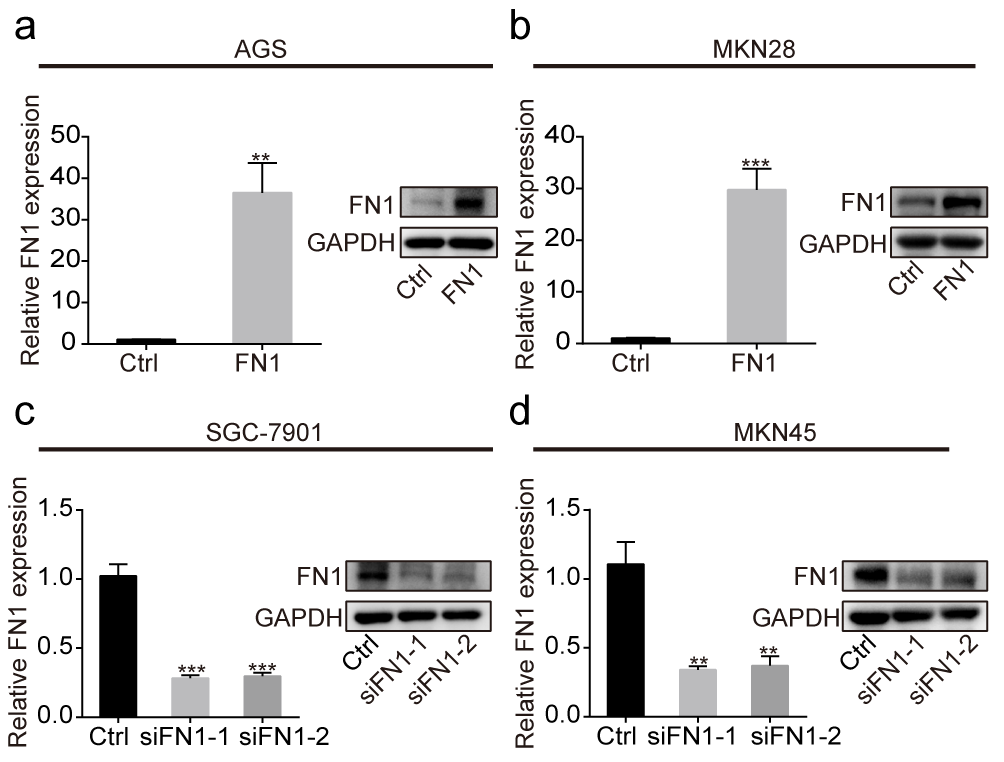

Supplement: Supplementary file 2 — Additional file 2: Figure S2. FN1 overexpression plasmid or siRNA targeting FN1 was transfected into GC cells. a, b AGS and MKN28 cells were transfected with FN1 overexpression plasmid. c, d SGC-7901 and MKN45 cells were transfected with siFN1-1 and siFN1-2 (**p < 0.01, ***p < 0.001). [file 40164_2022_260_MOESM2_ESM.tif]
