# Supplementary material for: HOXA13 promotes gastric cancer progression partially via the FN1-mediated FAK/Src axis
Source: Exp Hematol Oncol. 2022 Feb 23;11:7. doi: 10.1186/s40164-022-00260-7 (PMC8864865; doi:10.1186/s40164-022-00260-7)
Supplement: Supplementary file 3 — Additional file 3: Table S1. Primer sequences for qRT-PCR. Table S2. Primary antibodies for Western blot analysis. Table S3. Primer sequences for ChIP assay. [file 40164_2022_260_MOESM3_ESM.docx]

**Additional file 3: Tables S1** Primer sequences for qRT–PCR

| Gene | Primer sequences |
| --- | --- |
| HOXA13 | Forward: 5′-GAACGGCCAAATGTACTGCC-3′ |
|  | Reverse: 5′-GTATAAGGCACGCGCTTCTTTC-3′ |
| FN1 | Forward: 5′-CCAACTTCCTGGTGCGTTACTCACC-3′ |
|  | Reverse: 5′-CGTAGACACTGGAGACACTCACTACA-3′ |
| GAPDH | Forward: 5′-GGGAAGGTGAAGGTCGGAGT-3′ |
|  | Reverse: 5′-GGGGTCATTGATGGCAACA-3′ |
| miR-449a | Forward: 5′-CGCGTGGCAGTGTATTGTTA-3′ |
|  | Stem loop primer: 5′-GTCGTATCCAGTGCAGGGTCCGAGGTATTCGCACTGGATACGACACCAGC-3′ |
|  | Universal reverse primer: 5′-AGTGCAGGGTCCGAGGTATT-3′ |
| U6 | Forward: 5′-GCTCGCTTCGGCAGCACATATAC-3′ |
|  | Stem loop primer: 5′-GTCGTATCCAGTGCAGGGTCCGAGGTATTCGCACTGGATACGACAAAAATATGG-3′ |
|  | Universal reverse primer: 5′-AGTGCAGGGTCCGAGGTATT-3′ |

**Additional file 3: Tables S2** Primary antibodies for Western blot analysis

| Antibody | Concentration | Company |
| --- | --- | --- |
| HOXA13 | 1:1000 | Abcam, Cambridge, UK |
| FN1 | 1:1000 | Cell Signaling Technology, Beverly, MA, USA |
| N-cadherin | 1:1000 | Cell Signaling Technology |
| E-cadherin | 1:1000 | Cell Signaling Technology |
| MMP9 | 1:1000 | Cell Signaling Technology |
| Vimentin | 1:1000 | Cell Signaling Technology |
| p-FAK (Tyr397) | 1:1000 | Cell Signaling Technology |
| FAK | 1:1000 | Cell Signaling Technology |
| p-Src (Tyr416) | 1:1000 | Cell Signaling Technology |
| Src | 1:1000 | Cell Signaling Technology |
| p-Erk1/2 | 1:1000 | Cell Signaling Technology |
| Erk1/2 | 1:1000 | Cell Signaling Technology |
| Bcl2 | 1:1000 | Cell Signaling Technology |
| Cleaved PARP | 1:1000 | Cell Signaling Technology |
| Cleaved Caspase-9 | 1:1000 | Santa Cruz, Dallas, Texas, USA |
| Cleaved Caspase-3 | 1:1000 | Santa Cruz |
| Bax | 1:1000 | Santa Cruz |
| p-Akt | 1:1000 | Santa Cruz |
| Akt | 1:1000 | Santa Cruz |
| ITGB1 | 1:1000 | Proteintech, Hubei, China |
| ITGA5 | 1:1000 | Abcepta, Jiangsu, China |
| GAPDH | 1:1000 | Abcepta |

**Additional file 3: Tables S3** Primer sequences for ChIP assay

|  | Primer sequences |
| --- | --- |
| P1 | Forward: 5′-TGGAAGAAACACTCGGCTTC-3′ |
|  | Reverse: 5′-TCTTTGTGACCCACACTCCA-3′ |
| P2 | Forward: 5′-ATTTGCTGGGTGTTTTGCTT-3′ |
|  | Reverse: 5′-CCTTCAGTAATTGCCACAGGA-3′ |
| P3 | Forward: 5′-CTGAACTTCCCCGGGATCTG-3′ |
|  | Reverse: 5′-TCTTCACAGCTCCCTGTTCG-3′ |
| P4 | Forward: 5′-AGAGAGGTGACGCAATGTCC-3′ |
|  | Reverse: 5′-TGGACTTGTGTGAAGCGAAG-3′ |
| P5 | Forward: 5′-TTCGCTTCACACAAGTCCAG-3′ |
|  | Reverse: 5′-CCTTTGCGGTCATCAAACTT-3′ |
| P6 | Forward: 5′-GGACCGTCCCATATAAGCCC-3′ |
|  | Reverse: 5′-CAGAAGGGATGCAGAGGACC-3′ |
